# Changes in incidence of rotablation-induced coronary perforation by modifying practice over time: experiences at a high-volume center

**DOI:** 10.3389/fcvm.2026.1895205

**Published:** 2026-08-20

**Authors:** Hsiao-Tse Tu, Yu-Hsiang Wang, Yen-Po Lin, Tian-Chien Tsai, Ming-Ju Chuang, Yu-Wei Chen, Chi-Wei Wang, Chieh-Shou Su, Chi-Yan Wang, Tsun-Jui Liu, Kae-Woei Liang, Yun-Yu Chen, Wen-Lieng Lee

**Affiliations:** 1 Cardiovascular Center, Taichung Veterans General Hospital, Taichung, Taiwan; 2 Institute of Emergency and Critical Care Medicine, National Yang Ming Chiao Tung University School of Medicine, Taipei, Taiwan; 3 Institute of Clinical Medicine, National Yang Ming Chiao Tung University School of Medicine, Taipei, Taiwan; 4 Department of Medicine, National Yang Ming Chiao Tung University School of Medicine, Taipei, Taiwan; 5 Institute of Medicine, Chung-Shan University School of Medicine, Taichung, Taiwan; 6 Division of Cardiology, Department of Medicine, Pingtung Veterans General Hospital, Pingtung, Taiwan; 7 Department of Medical Research, Taichung Veterans General Hospital, Taichung, Taiwan; 8 Department of Post-Baccalaureate Medicine, National Chung Hsing University School of Medicine, Taichung, Taiwan

**Keywords:** bias cutting, complex and higher-risk percutaneous coronary intervention, coronary perforation, rotational atherectomy (RA), structured education

## Abstract

**Background:**

Rotational atherectomy (RA)-induced coronary perforation (CP) represents a dreaded complication, with incidence rates remaining constant and non-negligible for patient safety.

**Objective:**

In 2020, we published a paper on four mechanisms of RA-induced CP. In this paper, we hypothesized that modifying practice through general awareness of the mechanisms of RA-induced CP and providing mentorship could lead to a reduction in the incidence of CP.

**Methods:**

Consecutive RA patients were recruited and stratified into two groups: January 2009 to December 2019 (Period I) and January 2020 to February 2024 (Period II). During Period II, efforts (structured education, case advice, and case review) to promote general awareness of the four mechanisms of the RA-induced CP were made and mentorship from experienced operators was provided to ensure adherence to good RA practice.

**Results:**

A total of 877 patients were recruited: 510 in Period I and 367 in Period II. Most RA procedures were performed for single vessels in both periods. For non-left main vessels, more distally located and bifurcation lesions were treated in Period I, but the incidence of severe calcification was slightly higher in Period II than in Period 1. Less RA was performed for side branches only, and the use of only the 1.25 mm burr increased in Period II. RA could be successfully completed in a high percentage of patients in both periods (98.4% vs. 98.4%, *p* = 0.938). The final stent size and total stent length were similar in the two periods. Baseline and gain in Syntax scores were higher in Period I than in Period II, but the residual Syntax scores were similar. More patients were prophylactically put on mechanical circulatory support for CHIP in Period II. Acute slow/no flow (4.9% vs. 9.0%, *p* = 0.021), RA-induced CP (0.3% vs. 2.0%, *p* = 0.027), and acute heart failure/pulmonary edema (0.3% vs. 2.9%, *p* = 0.004) were significantly reduced in Period II. No CP events occurred secondary to wire tip injury, wire transection, burr oversizing, or bias cutting in Period II.

**Conclusion:**

Modifying RA practice through general awareness of the four mechanisms of RA-induced CP and provision of mentorship from experienced operators helped lower the incidence of this fearful complication.

## 1 Introduction

Percutaneous coronary intervention (PCI) has become a well-recognized modality for treating coronary artery disease (CAD), including high-risk complex lesions (1), particularly when surgical revascularization is not considered because of multiple comorbidities and high risks (2). However, heavy calciﬁcation remains a formidable challenge to PCI, even for well-experienced operators (3). Heavy calciﬁcation could hinder adequate preparation of the vessels, prevent device delivery, or cause stent under-expansion or even dislodgement, resulting in acute adverse events in catheterization laboratories (4). Long-term clinical outcomes of stent performance secondary to inadequate calciﬁed lesion preparation could also be suboptimal (5). Heavy calciﬁcation may be eccentric, bulky, very long with multiple acute bends, and encountered in acute coronary syndrome. Although intravascular lithotripsy (IVL) and orbital atherectomy (OA) are alternatives to treat coronary calciﬁcation (6), rotational atherectomy (RA) has been employed to treat coronary calcification for a long time, is recognized as the standard treatment modality, and remains familiar to most interventionists. Despite its efficacy, high success rates, and low complication rates, the rate of incidence of RA-related coronary perforation (CP) has remained stable at 0.4%–3.5% over the years (7, 8), even in experienced centers and in experienced hands (9, 10). This complication should not to be ignored given its grave consequences once it occurs. In recent years, high-risk indicated PCI procedures (CHIPs) have become a hot topic, often employed to treat complex and heavily calcified lesions (11). In such circumstances, CP carries severe morbidity and mortality. In 2020, our group published a paper on the mechanisms of RA-induced CP, identifying four mechanisms by reviewing all CP cases across 9 years at our catheterization laboratory (12). Among the four mechanisms, the most important but often overlooked was unintended and unnoticed RA bias cutting, which accounted for 60% of all cases. This mechanism was well illustrated in diagrams in our prior paper. Following that work, structured education on these mechanisms and strategies for prevention of CP were enthusiastically promoted and reiterated at our catheterization laboratory and in our meetings. General awareness of these mechanisms and with heightened operator cautiousness may be key elements in evading these complications. We hypothesized that modification of RA practice through structured efforts (structured education, case advice, and case review) to promote general awareness of the four mechanisms of the RA-induced CP, together with mentorship from experienced operators to less experienced ones, particularly during the early phases of their learning curve, could lead to a reduction in the incidence of this dreaded complication and improve procedural outcomes. This benefit may be underestimated and deserves emphasis. Accordingly, the present study was designed to explore the temporal changes in the incidence of RA-related CP after the publication of our previous paper and confirm our hypothesis.

## 2 Methods

The RA protocol was basically the same as that stated in our previous papers, which is still abided by our colleagues at the catheterization laboratory (12–14). This RA practice was modified in Period II.

### 2.1 Patient population

This was a retrospective study designed to evaluate any improvement in procedural RA performance. Consecutive patients who underwent RA for calcified coronary lesions between January 2009 and February 2024 were identified from the catheterization laboratory database and by manual inspection. Indications for PCI, procedural details, and any complication at the time of the index PCI were retrieved and recorded. Electronic medical records for each patient were also reviewed in detail, and relevant clinical information and biochemical ﬁndings at the time of hospitalization were retrieved and recorded in the case record form. Patients were stratiﬁed into two groups according to the publication date of our reference paper: January 2009 to December 2019 (Period I, pre-publication) and January 2020 to February 2024 (Period II, post-publication).

### 2.2 RA, angiographic characterization, and measurements

A workstation with dedicated software (Rubo DICOM Viewer, Version 2.0, Build 170828, Rubo Medical Imaging, Aerdenhout, The Netherlands) was used to review the coronary angiograms and make quantitative measurements. SYNTAX scores before and after PCI were calculated using the standard online calculator software. The number of diseased coronary arteries was defined as the total of the three major coronary vessels with stenosis ≥70% in diameter and the left main lesion was defined as ≥50% diameter stenotic. Severe coronary artery calciﬁcation was deﬁned as the presence of apparent abluminal radio-opacity along the sides of the vascular walls, appearing in two different cine projections without cardiac movement and before the injection of contrast medium.

All PCI procedures were performed by certiﬁed interventional cardiologists in our catheterization laboratory, in accordance with standard practice. Patients were pretreated with a standard dose of aspirin and clopidogrel (or prasugrel/ ticagrelor as indicated). Calcium channel blockers and nitrates were also used to prevent coronary artery spasm. Heparin was administered to maintain an activated clotting time of ≥300 s during the procedure. The decision to perform RA was based on standard practice and operator discretion. The indications for RA could be primary (for heavy and circular/rotating intimal calciﬁcation) or secondary as bail-out procedure (for device undilatable or uncrossable lesions). Prior to RA, a 0.009 in. ﬂoppy or extrasupport RotaWire™ with a gently J-curved tip was advanced by the operator through the lesion using a microcatheter. A bolus of 1,200–1,600 µg of isosorbide dinitrate was administered intracoronarily prior to the start of RA, during which normal saline mixed with heparin and isosorbide dinitrate was slowly infused. RA was performed using the Rotablator™ (from the beginning) or ROTAPRO™ RA system (merged since 2022) (RotaLink Plus), starting with a 1.25 (for most bail-out indications and vessels with very small lumen) or 1.5 mm burr at a speed of 170,000–180,000 rpm. This was often supplemented by another burr that was one size bigger. Each burr advancement lasted less than 20–30 s, while polishing times after burr passing varied and depended on operator discretion. For patients who needed side branch (SB) rotablation, the sequence of RA of SB or main vessel (MV) treatment was determined by the criterion of which vessel was more critically diseased and would be potentially jeopardized if not treated ﬁrst; operator discretion was also taken into account. After the vessel and blood ﬂow in the ﬁrst treated vessel were secured, rotawiring of the second vessel was performed, followed by RA. After RA was accomplished, the RotaWire™ was replaced by a workhorse wire through a microcatheter, and the procedure was continued with balloon angioplasty with or without stent implantation. Completion of RA was deﬁned as intended debulking of the target lesion without premature termination of RA. After stent implantation, dual antiplatelet therapy with aspirin (100 mg/day) and clopidogrel (75 mg/day; or Prasugrel 3.75 mg/day, or ticagrelor 90 mg twice a day as indicated) was continued according to standard practice.

**Figure 1:**
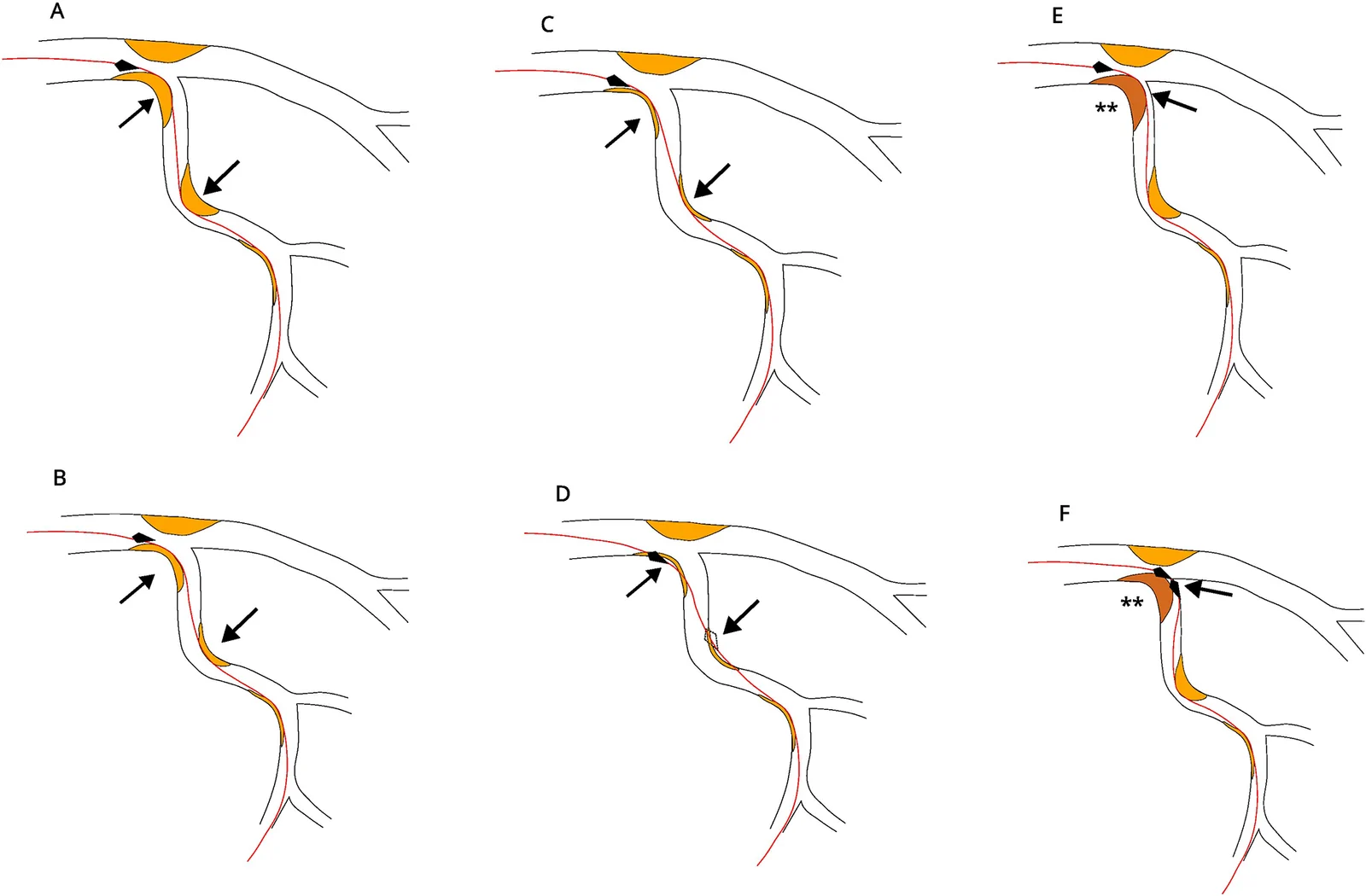
Favorable bias cutting and exception lesions. RA can be utilized to ablate thick calcium plates or calcified nodules located at the inner corner of acute bends **(**arrows, panel **A)**. Using normal frame rate, normal field of vision (FOV), and fluoroscopic setting is encouraged, with careful attention to the relationship between the burr/wire and the vessel wall calcium. During the procedure, thinning of the calcium plate by RA can be observed **(**arrows, panel **B)**. The operator should stop further rotablation when a majority of the calcium is sequentially taken out by the burr and a minimal amount of calcium is left **(**arrows, panel **C)**. Overdoing rotablation could lead to the burr cutting through the vessel wall and perforating the vessel **(**arrows, panel **D)**. However, in extreme cases, the calcium chunk could be too thick, solid, and hard to be taken out by RA (**, panel **E)**. In this case, the burr could slide over the calcium chunk and cut into the outer corner of the downstream acute bend **(**arrow, panel **E**) and precipitate vessel perforation **(**arrow, panel **F)**.

**Figure 2:**
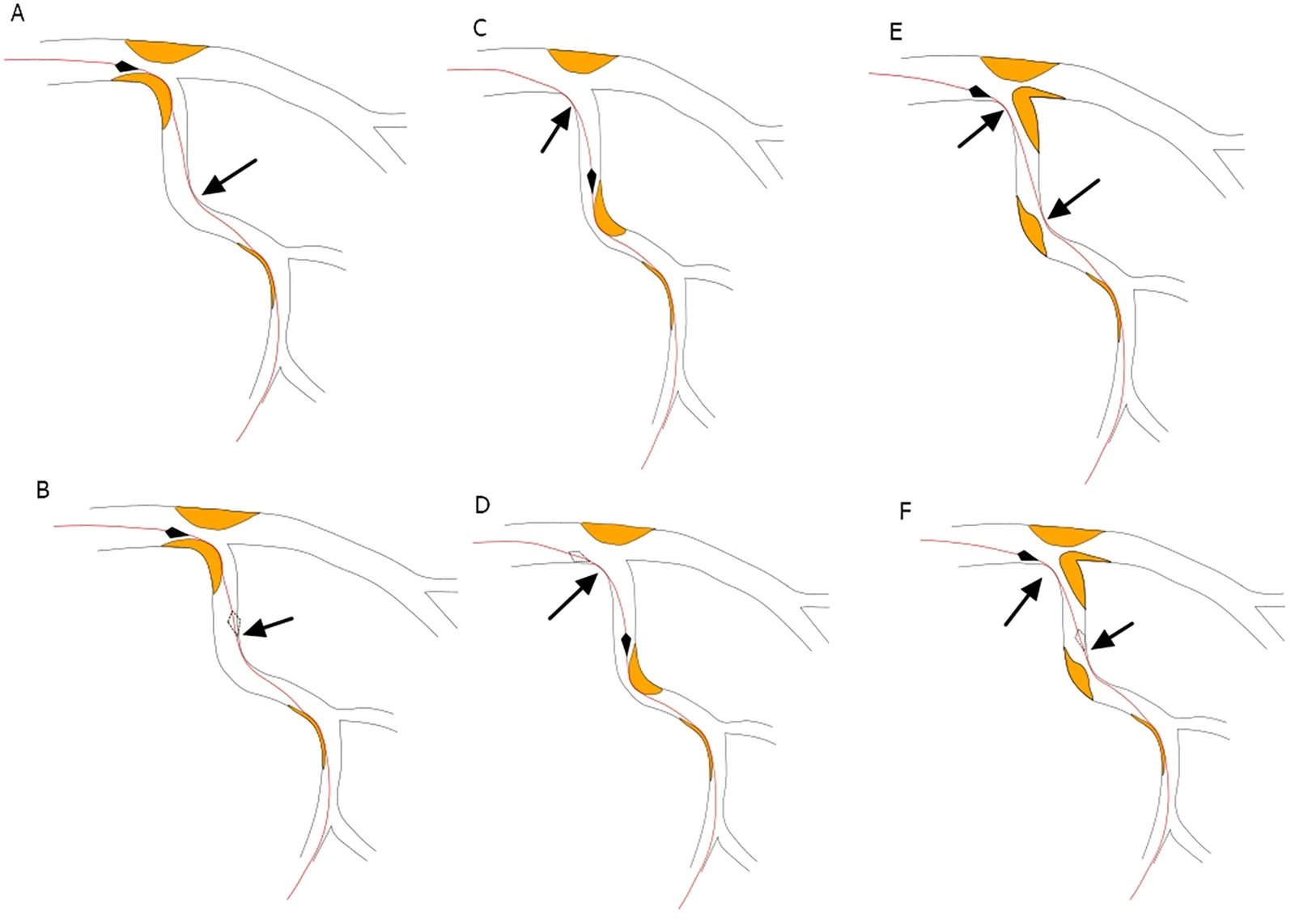
Unfavorable RA bias cutting. When RA bias cutting is utilized to treat thick calcium at inner corners of acute bends, the burr may go too far down and reach the calcification-free inner corner of the downstream bend **(**arrow, panel **A)** or travel though a similar lesion at an upstream bend **(**arrow, panel **C)** before it reaches the intended target lesion. Because there is no calcium at those sites, unfavorable bias cutting occurs, potentially leading to vessel perforation at these sites **(**arrows, panels **B,D)**. Unfavorable bias cutting also occurs when RA is intended to treat calcified lesions located at the outer corners of acute bends, where the burr can easily cut through the non-calcified inner corners, leading to perforation **(**arrows, panels **E,F)**.

**Figure 3:**
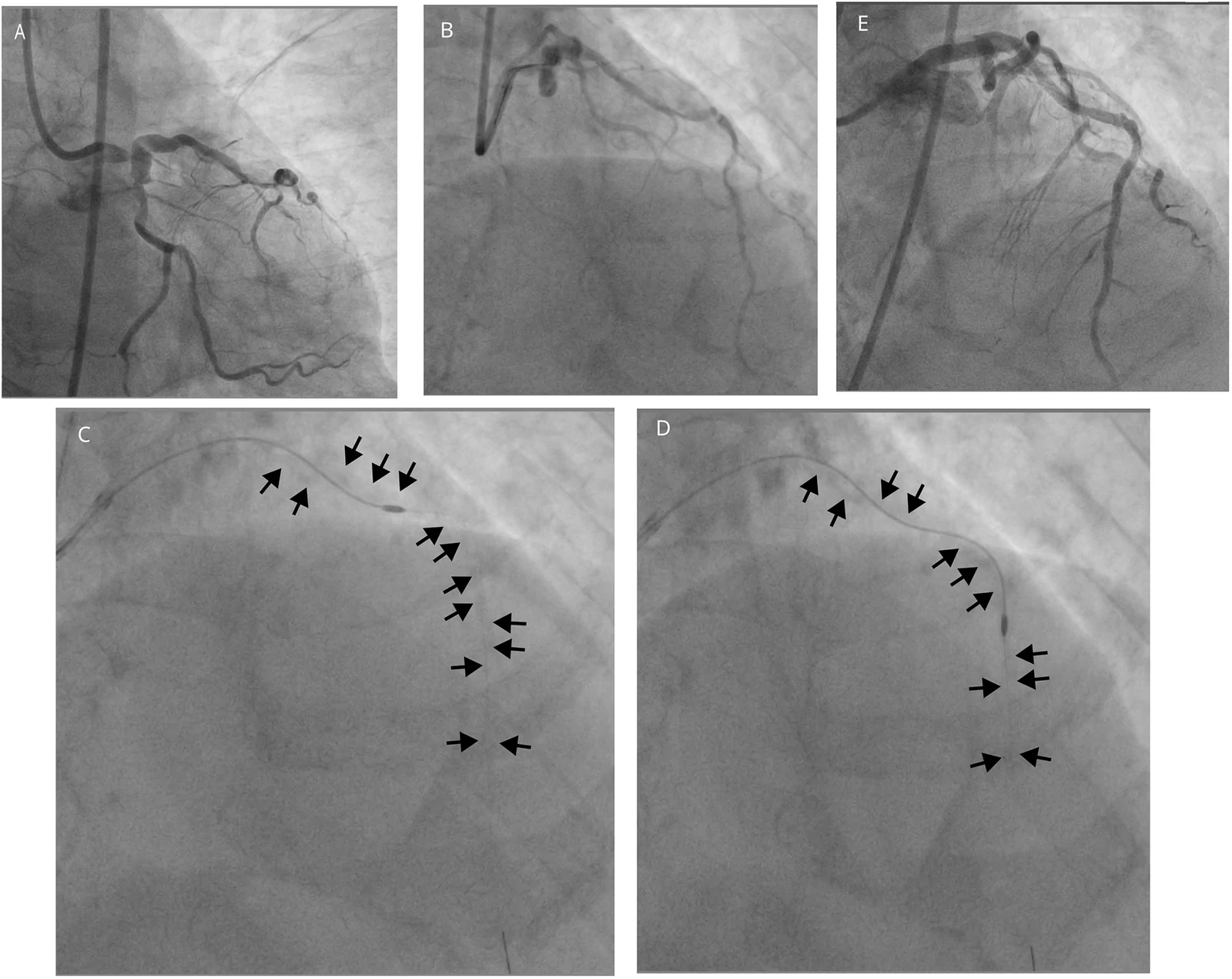
An example case of complex and heavily-calcified lesions which could be treated by RA using favorable bias cutting. The calcified lesions involved LM bifurcation, ostial-proximal-middle-distal LAD **(**panels **A,B)** and proximal LCX **(**panel **A)**, which was well treated with rotablation plus DES for all above lesions as the patient definitely defied CABG. Given all the thick calcifications were located at inner corners of curvatures (black arrows) which were best appreciated when the extrasupport RA wire was in place, they were RA bias cutting favorable and comfortably treated **(**panels **C,D)**, leading to well acceptable angiographic results **(**panel **E)**.

**Figure 4:**
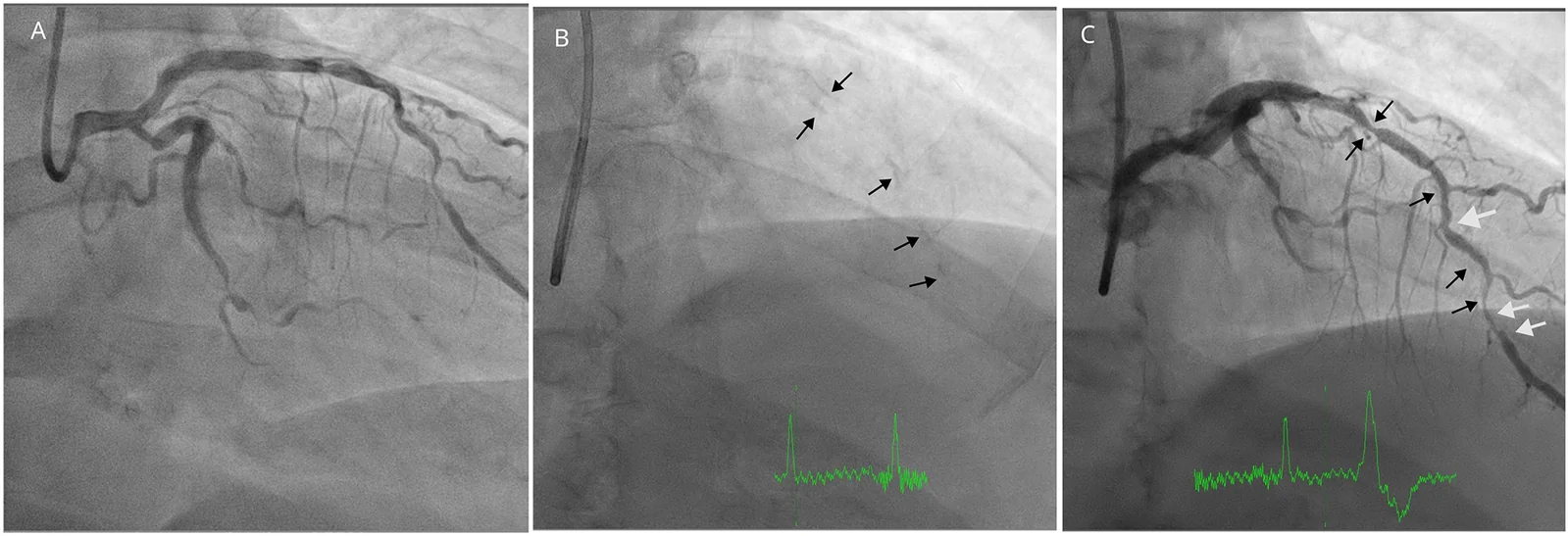
A patient with heavily calcified CAD involving the distal LM bifurcation, mid-distal LAD, and proximal LCX **(**panel **A)**. Thick calcification was eccentric in distribution and located at the outer corners of acute bends in the distal LAD **(**black arrows, panels **B,C)**, while the inner corners of the corresponding bends were free of thick calcium and prone to RA bias cutting through the vessel wall **(**white arrows, panel **C)**. Because of safety reasons, RA was not considered and CABG was chosen instead.

**Figure 5:**
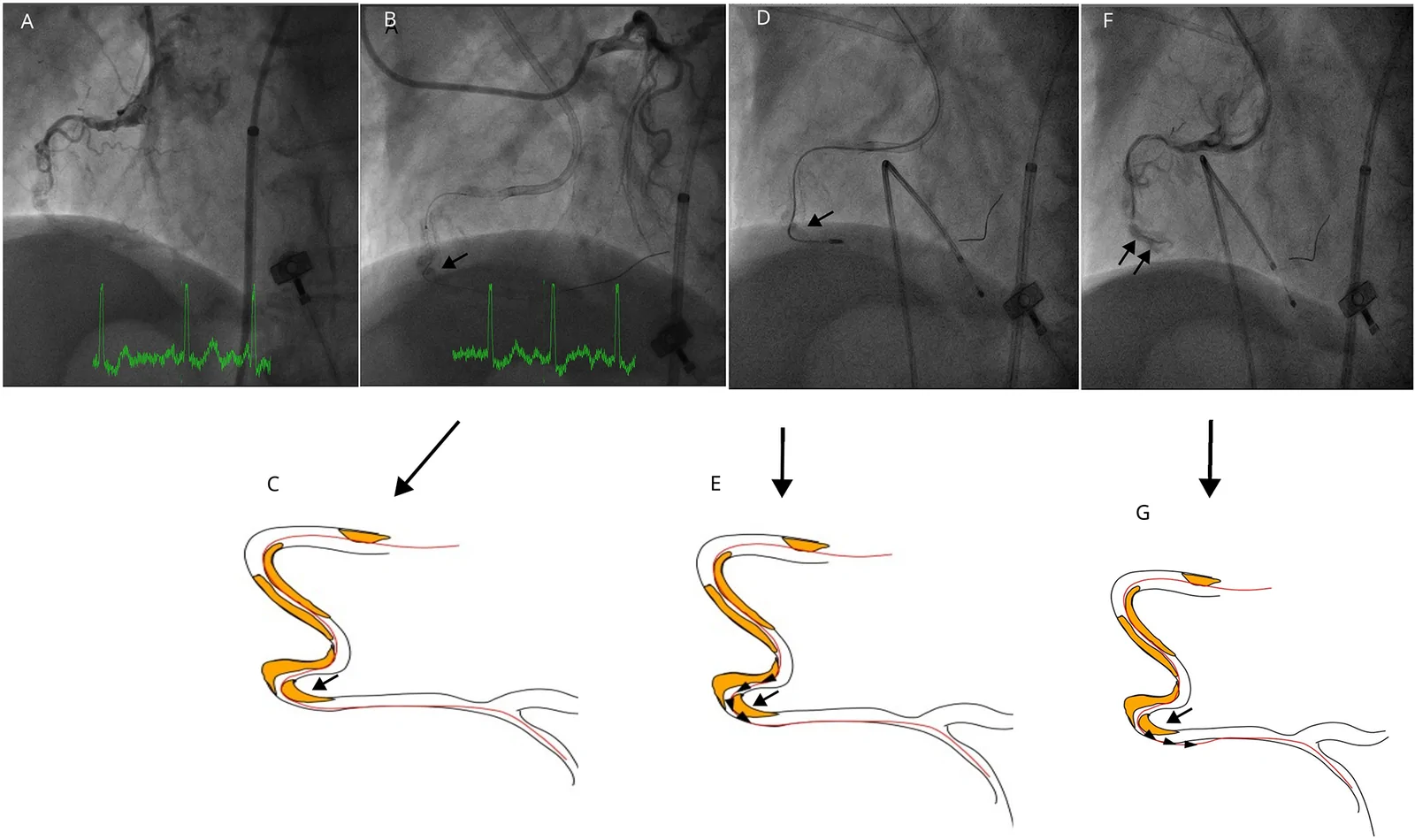
A patient with RA-induced CP at distal RCA. The patient had occlusive ISR at mid-RCA, followed by a CTO lesion **(**panel **A)**. After the wire was passed, no device could be advanced. Therefore, RA was considered and the wire was placed. However, there was acute angulation with a small radius at the mid-distal junction of the RCA and a large/thick calcium at the inner corner **(**arrows, panels **B**,**C)**. The RA burr could not ablate the thick calcium but slid over it to a downstream segment **(**arrowheads, panels **D,E)** and bias cut through the non-calcified vessel wall after multiple RA runs, leading to perforation **(**arrows, panel **F**, and arrowheads, panel **G)**. This mechanism is explained in panels **E,F** of Figure 1.

During Period II, structured education efforts (case advice and case review) were made to promote general awareness of the four mechanisms of RA-induced CP and to modify rotablation practice in order to avoid CPs. Key strategies included maintaining the wire within the visible distal branch in order to prevent perforation of the tiny end vessel, avoiding rotablation in very small vessels (<2 mm) where even the smallest burr may cut through the vessel wall at acute bends, using extrasupport RotaWire when strong support was needed so that wire transection could be avoided, and taking advantage of favorable bias cutting, while avoiding unfavorable bias cutting, as illustrated in Figures 1, 2. After diagnostic angiography, cine images were reviewed in detail till the location and distribution of calcium along the vessel tree were confidently defined, which were best appreciated when the RotaWire was in place. The key points of using favorable RA bias cutting are illustrated in Figure 1. RA bias cutting could be utilized to ablate thick calcium plates or calcified nodules located at the inner corner of acute bends (arrows, panel A). The use of a normal frame rate (15 fps), a normal field of vision (FOV) but not low magnification, and a regular but not low-radiation fluoroscopic setting is encouraged. Under these conditions, great care must be exerted to observe the calcium plate thickness and relationship between burr/wire and vessel wall calcium. During the procedure, thinning of the calcium plate by RA could be observed (arrows, panel B). The operator was advised to stop further rotablation when the majority of the calcium had been sequentially taken out by burr and only a minimal amount of calcium was left over (arrows, panel C). Overdoing rotablation could lead to the burr cutting through the vessel wall and perforating the vessel (arrows, panel D). However, in extreme cases, the calcium chunk could be very thick, solid, and hard to take out (**, panel E). In this case, the burr could slide over the calcium chunk and cut into the outer corner of the acute bend (arrow, panel E) and precipitate vessel perforation (arrow, panel F). Lesions unfavorable for RA bias cutting are illustrated in Figure 2. When RA bias cutting was utilized to treat thick calcium at the inner corners of acute bends, the burr could advance too far down and reach the calcification-free inner corner of the downstream bend (arrow, panel A) or travel though a similar lesion at an upstream bend (arrow, panel C) before reaching the intended target lesion. Because there is no calcium at those sites, unfavorable bias cutting occurs and too much ignorance could lead to vessel perforation at these sites (arrows, panels B and D). Unfavorable bias cutting also occurred when RA was directed at calcified lesions located at the outer corners of acute bends, easily cutting through the non-calcified inner corners, leading to perforation (arrows, panels E and F). A typical case of complex, high-risk CAD with heavily calcified lesions involving distal LM, LCX, and long segments of LAD was successfully treated with RA across all lesions using favorable bias cutting, as shown in Figure 3. Another similar case with heavily calcified lesions in the distal LM, LCX, and long segments of LAD were deemed not suitable for RA because of unfavorable bias cutting, as shown in Figure 4. At the opposite end of the spectrum, a case of RA-related CP at the distal right coronary artery (RCA) caused by bias cutting is shown in Figure 5, with the mechanism corresponding to panels E and F of Figure 1.

**Figure 6:**
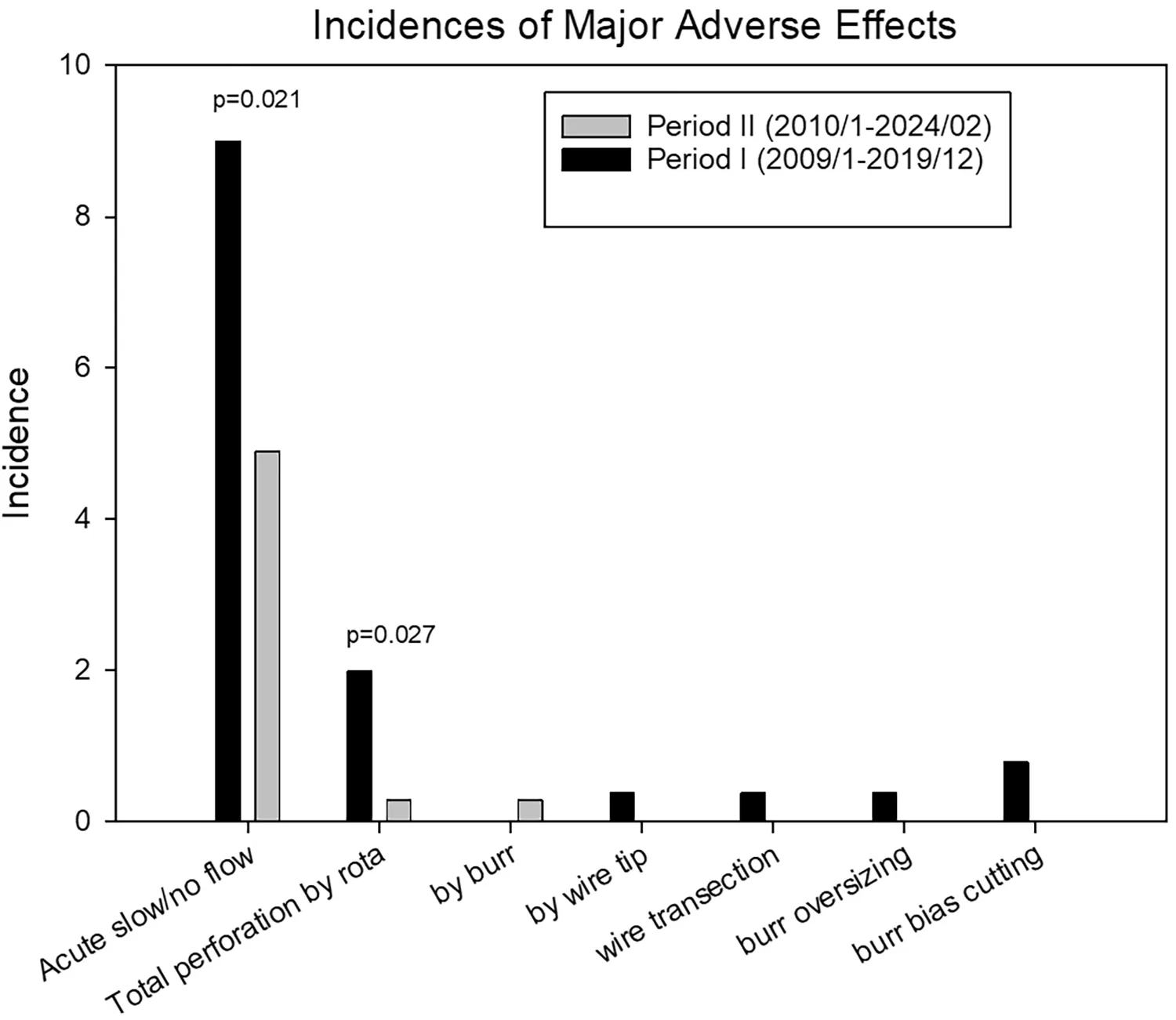
Incidences of major adverse effects during RA. There was a significant reduction in the incidence of acute slow/no flow and total RA-induced CP in Period II.

During the structured education sessions, we demonstrated how we identified the four mechanisms by reviewing all the CP case cine recordings in detail. We dissected the cines run by run to illustrate each mechanism. This was very helpful, especially in those cases where the wrongdoing was subtle and difficult to catch at first sight. Case mentorship and support from experienced RA operators at the catheterization room was also provided to help trainees recognize the distribution of calcium along the vessel wall, particularly during the early phases of their learning curve. Mentorship was also provided to help perform RA bias cutting in the correct way, thereby promoting good practice.

As orthogonal fluoroscopic projections are more advantageous for identifying the distribution of calcification and its relationship to rotablation wire/burr during real-time intervention, our techniques were developed in the traditional way, without intravascular imaging (IVI). Furthermore, single two-dimensional cross-sectional images of the vessel by IVI cannot reflect the real-time four-dimensional relationship between the RA burr and the vessel wall calcium. In order to better control burr behavior, the advancing speed of the burr was kept low and gentle force was applied during RA.

Major complications occurring during RA were retrieved and recorded, including the incidence of coronary perforations. The underlying cause of each CP was identified by reviewing the session’s cine recordings in detail. In case of complications during the procedure, diagnosis and emergent management were performed according to standardized protocols tailored to the speciﬁc complication. The reasons for incomplete rotablation and the need for hemodynamic support during the procedure were also carefully examined from the catheterization laboratory database.

### 2.3 Statistics

Categorical data were expressed in terms of number and frequency. Continuous variables were presented as mean ± standard error. Differences in categorical data were analyzed using the chi-square test, and differences in continuous variables were measured by the unpaired Student's *t*-test. As the study population consisted of consecutive patients with different clinical spectra, some variables were not normally distributed, for which an independent-sample Mann–Whitney *U* test was used. All statistical analyses were performed using IBM SPSS Statistical Software for Microsoft Windows, Version 27.0 (IBM Corp., New York, USA). Two-tailed *p* values below 0.05 were considered statistically signiﬁcant.

## 3 Results

### 3.1 Patient characteristics

A total of 877 patients were recruited in the study. Of these, 510 patients (334 males/176 females) belonged to Period I, while 367 patients (261 males/106 females) belonged to Period II. Patients in Period II were significantly younger than those in Period I [70.8 ± 0.6 (mean ± SEM) vs. 73.3 ± 0.5 years, *p* < 0.001; Table 1] and had more stable angina (45.2% vs. 25.3%). They also had lower rates of hypertension (44.4% vs. 63.3%, *p* < 0.001) and diabetes (45.5% vs. 56.1%, *p* = 0.002). There was no significant difference in the incidence of ischemic cardiomyopathy (CM), cardiogenic shock, chronic kidney disease (CKD), peripheral artery disease, prior coronary artery bypass grafting (CABG), or left ventricular ejection fraction between the two groups. However, Period II patients had fewer single-vessel diseases but more multiple-vessel diseases (Table 1).

**Table 1:** Patient characteristics and laboratory findings.

	Period I	Period II	*p*-value
	(2009/1 to 2019/12)	(2020/1 to 2024/2)	
Variables	*N* = 510	*N* = 367	
Sex (M/F)	334/176	261/106	0.078
Age (years)	73.3 ± 0.5	70.8 ± 0.6	<0.001
Clinical diagnosis (*N*, %)			<0.001
Stable angina	129 (25.3)	166 (45.2)	
Unstable angina	170 (33.3)	71 (19.3)	
NSTEMI	116 (22.7)	62 (16.9)	
STEMI	25 (4.9)	14 (3.8)	
Ischemic CM	70 (13.7)	54 (14.7)	
Cardiogenic shock	26 (5.1)	14 (3.8)	0.368
Hypertension (*N*, %)	323 (63.3)	163 (44.4)	<0.001
Diabetes (*N*, %)	286 (56.1)	167 (45.5)	0.002
Dyslipidemia (*N*,%)	158 (31.0)	97 (26.4)	0.143
PAD (*N*, %)	46 (9.0)	23 (6.3)	0.135
CKD (*N*, %)	162 (31.8)	105 (28.6)	0.317
Af/AF (*N*, %)	29 (5.7)	27 (7.4)	0.318
History of CABG (*N*,%)	52 (10.2)	50 (13.6)	0.118
Baseline LVEF (%)	46.2 ± 0.7	46.7 ± 0.8	0.604
Lab data			
Hemoglobin (mg/dL)	11.2 ± 0.1	11.7 ± 0.1	< 0.001
BUN (mg/dL)	28 (6–124)	27 (5–143)	0.925
CR (mg/dL)	1.3 (0.3–15.6)	1.4 (0.5–14.8)	0.397
EPI-eGFR (mL/min)	50.4 (3.4–111.2)	49.8 (3.4–158.4)	0.753
HDL-chol (mg/dL)	44.0 ± 0.7	44.4 ± 0.9	0.689
LDL-chol (mg/dL)	82.9 ± 1.6	74.6 ± 1.9	< 0.001
FBS (mg/dL)	150 ± 5	149 ± 5	0.855
HbA1c (mg/dL)	6.7 ± 0.1	6.7 ± 0.1	0.960
Total CK (U/L)	255 ± 38	223 ± 28	0.528
CK-MB (U/L)	14.6 ± 2.1	9.6 ± 0.9	0.028
Troponin-T (ng/mL)	40.2 (0.0–10,000)	57.6 (4.0–8,240)	0.457
CAD vessel numbers			
SVD (*N*, %)	181 (35.5)	87 (23.7)	<0.001
DVD (*N*, %)	139 (27.3)	140 (38.1)	
TVD (*N*, %)	190 (37.3)	140 (38.1)	
LM (*N*, %)	68 (13.3)	33 (9.0)	0.047
Isolated	0	4 (1.1)	
Plus	68 (13.3)	29 (7.9)	

Continuous variables are expressed as mean ± SEM; BUN, Cr, EPI-eGFR, and troponin-T are expressed as median (range). Af/AF, atrial fibrillation or flutter; CABG, coronary artery bypass grafting; CAD, coronary artery disease; CM, cardiomyopathy; CR, creatinine; CKD, chronic renal disease; DVD, double-vessel disease; ESRD, end-stage renal disease; FBS, fasting blood sugar; LM, left main coronary artery; LVEF, left ventricular ejection fraction; NSTEMI, non-ST elevation myocardial infarction; PAD, peripheral artery disease; STEMI, ST-elevation myocardial infarction; SVD, single-vessel disease; TVD, triple-vessel disease.

Patients in Period II had higher hemoglobin levels (11.7 ± 0.1 vs. 11.2 ± 0.1, *p* < 0.001) and lower LDL-C concentrations (74.6 ± 1.9 vs. 82.9 ± 1.6, *p* < 0.001). However, there were no significant differences between the groups in the levels of median serum creatine, EPI-eGFR, HbA1c, or baseline troponin-T.

### 3.2 Rotablation lesion characteristics and procedural details

The characteristics of rotablation-treated lesions and procedural details are summarized in Table 2. In both Periods I and II, most rotablation procedures were performed for single-vessel disease (82.9% vs. 83.7%), with the remainder involving two or three vessels (17.1% vs. 16.3%). There was no difference in the number of left main (LM) lesions treated between patients in Period I and those in Period II, except that more LM body lesions were treated in Period II patients (7.4% vs. 4.5%, *p* = 0.001). Among the non-left main vessels treated with rotablation, there were more distally located lesions (63.5% vs. 47.1%, *p* < 0.001) and bifurcation lesions (32.2% vs. 16.1%, *p* < 0.001) in Period I, but the incidence of heavy calcification was slightly higher in Period II (89.1% vs. 86.1%, *p* = 0.047). There was no difference in the incidence of chronic total occlusion between the two periods. Moreover, there was no difference in total lesion numbers, total lesion lengths, or incidence of ACC/AHA type C lesions between patients in Period I and Period II.

**Table 2:** Procedural details, RA vessels, and interventional characteristics.

	Period I	Period II	*p*-value
	(2009/1 to 2019/12)	(2020/1 to 2024/2)	
Variables	*N* = 510	*N* = 367	
Rotablation vessels			0.781
Single vessel disease, LM (*N*, %)	1 (0.2)	8 (2.2)	
LAD (*N*, %)	278 (54.5)	184 (50.1)	
LCX (*N*, %)	47 (9.2)	37 (10.1)	
RCA (*N*, %)	97 (19.0)	78 (21.3)	
Multiple-vessel disease (*N*, %)			
LM + LAD (*N*, %)	22 (4.3)	10 (2.7)	
LM + LCX (*N*, %)	8 (1.6)	1 (0.3)	
LM + RCA (*N*, %)	1 (0.2)	1 (0.3)	
LAD + LCX (*N*, %)	28 (5.5)	25 (6.8)	
LAD + RCA (*N*, %)	14 (2.7)	10 (2.7)	
LCX + RCA (*N*, %)	1 (0.2)	2 (0.5)	
LM + LAD + LCX (*N*, %)	7 (1.4)	10 (2.7)	
LM + LAD + RCA (*N*, %)	6 (1.2)	1 (0.3)	
Rotablation lesion characteristics			
Left main (*N*, %)	45 (8.8)	31 (8.4)	0.763
Ostial	10 (2.0)	6 (1.6)	0.763
Body	23 (4.5)	27 (7.4)	0.001
Distal	38 (7.5)	27 (7.4)	0.747
Calcification			
Mild–moderate	1 (0.2)	1 (0.3)	0.798
Severe	44 (8.6)	30 (8.2)	
Non-left main			
(lesion in any major vessel)			
Location, ostial (*N*, %)	177 (34.7)	108 (29.4)	0.434
Proximal (*N*, %)	374 (73.3)	280 (76.3)	0.198
Middle (*N*, %)	455 (89.0)	307 (83.7)	0.345
Distal (*N*, %)	324 (63.5)	173 (47.1)	<0.001
Bifurcation (*N*, %)	164 (32.2)	59 (16.1)	<0.001
Calcification (*N*, %)			
Mild–moderate	26 (5.1)	9 (1.6)	0.047
Severe	439 (86.1)		327 (89.1)
Chronic total occlusion (*N*, %)	64 (12.5)	55 (15.0)	0.152
Total lesion length (mm)	45.2 ± 1.2	42.1 ± 1.2	0.072
Total lesion numbers (*N*)	3.4 ± 0.1	3.4 ± 0.1	0.659
ACC/AHA lesion (*N*, %)			
B2	49 (9.6)	33 (9.0)	0.609
C	455 (89.2)	308 (83.9)	0.074
Access site			<0.001
Radial (*N*, %)	172 (33.7)	193 (52.6)	
Femoral (*N*, %)	325 (63.7)	170 (46.3)	
Brachial (*N*, %)	13 (2.5)	4 (1.1)	
Guide size			<0.001
6F (*N*, %)	185 (36.3)	206 (56.1)	
7F (*N*, %)	318 (62.4)	158 (43.1)	
8F (*N*, %)	7 (1.4)	3 (0.8)	
Main/side branch rotablation (*N*, %)		0.017	
Main vessel only	450 (88.2)	334 (91.0)	
Main + side branch	23 (4.5)	22 (6.0)	
Side branch only	37 (7.3)	11 (3.0)	
Maximum burr size			0.001
1.25 mm (*N*, %)	105 (20.6)	93 (25.3)	
1.5 mm (*N*, %)	289 (56.7)	170 (46.3)	
1.75 mm (*N*, %)	109 (21.4)	82 (22.3)	
2.0 mm (*N*, %)	6 (1.2)	20 (5.4)	
2.25 mm (*N*, %)	1 (0.2)	2 (0.5)	
Rotablation completed	502 (98.4)	361 (98.4)	0.938
Final devices (*N*, %)			<0.001
POBA (*N*,%)	32 (6.3)	21 (5.7)	
BMS (*N*, %)	103 (20.2)	36 (9.8)	
DES (*N*, %)	360 (70.6)	275 (74.9)	
DEB (*N*, %)	9 (1.8)	30 (8.2)	
Stent graft	2 (0.4)	0 (0)	
RA burr *per se*	0 (0)	1 (0.3)	
Aborted procedure	4 (0.8)	4 (1.1)	
Mean stent numbers	1.9 ± 0.0	1.8 ± 0.0	0.013
Mean stent size (mm)	3.2 ± 0.1	3.4 ± 0.2	0.424
Total stent length (mm)	54.2 ± 1.3	55.9 ± 1.6	0.42
Syntax score			
Baseline	30.0 ± 0.6	25.1 ± 0.6	<0.001
Post-PCI	8.5 ± 0.5	7.1 ± 0.5	0.05
Gain	21.5 ± 0.5	18.0 ± 0.6	<0.001
Total procedure time (min)	164.0 ± 12.0	166.0 ± 3.7	0.874
Total fluoro time (min)	46.8 ± 1.1	51.3 ± 1.6	0.018
Total contrast dose (mL)	189.7 ± 3.5	168.6 ± 3.8	<0.001
Hemodynamic support (*N*, %)			0.002
IABP (*N*, %)	72 (14.1)	54 (14.7)	
ECMO	1 (0.2)	10 (2.7)	
Impella	0 (0)	5 (1.4)	
iVAC2L	0 (0)	1 (0.3)	
Indications for hemodynamic support			<0.001
Shock at beginning of procedure	21 (4.1)	7 (1.9)	
Shock during procedure	5 (1.0)	1 (0.3)	
Shock due to complication	1 (0.2)	0 (0)	
Preventive due to CHIP (*N*, %)	45 (8.8)	62 (16.6)	
Hemodynamic support removed			<0.001
At cath. room	25 (34.7)	44 (62.9)	
At CCU	47 (65.3)	26 (37.1)	

BMS, bare-metal stent; DEB, drug-eluting balloon; DES, drug-eluting stent; ECMO, extracorporeal membranous oxygenator; IABP, intra-aortic balloon pumping; LAD, left anterior descending artery; LCX, left circumflex artery; LM, left main coronary artery; RCA, right coronary artery.

More patients in Period II underwent PCI via radial access (52.6% vs. 33.7%, *p* < 0.001) and used a 6F sheath (56.1% vs. 36.3%, *p* < 0.001). Less rotablation was done for side branch in Period II (3% vs. 7.3%). In terms of burr size, the smallest 1.25 and 2 mm burrs were used in Period II (25.3% vs. 20.6% and 5.4% vs. 1.2%), while the overall completion rate of rotablation remained equally high in both periods (98.4% vs. 98.4%, *p* = 0.938). In terms of the final treatment device, more drug-eluting stents (74.9% vs. 70.6%) and more drug-eluting balloons (8.2% vs. 1.8%) were used in Period II. Despite fewer stents being used in Period II, the final stent size and total stent length were similar between Period I and Period II. Baseline (30.0 ± 0.6 vs. 25.1 ± 0.6, *p* < 0.001) and gain (21.5 ± 0.5 vs. 18.0 ± 0.6, *p* < 0.001) in Syntax scores were higher in Period I, but residual Syntax scores were almost similar (8.5 ± 0.5 vs. 7.1 ± 0.5, *p* = 0.05).

The total procedure time was similar between the two periods; however, the total fluoroscopy time was longer (51.3 ± 1.6 vs. 46.8 ± 1.1 min, *p* = 0.018) in Period II with less contrast medium (168.6 ± 3.8 vs. 189.7 ± 3.5 mL, *p* < 0.001) used. With regard to mechanical hemodynamic support (MCS) devices, more patients were put on these devices in Period II (19.1% vs. 14.3%, *p* = 0.002) and a majority of them were used prophylactically in CHIP (16.6% vs. 8.8%). More patients were weaned off the MCS at the catheterization laboratory (62.9% vs. 34.7%, *p* < 0.001).

### 3.3 Incidence of perforation, other adverse effects, and reasons for rotablation failure

The incidences of major adverse effects, coronary perforation, and rotablation failure are summarized in Table 3 and Figure 6. Compared with Period I, occurrences of acute slow/no flow (4.9% vs. 9.0%, *p* = 0.021), RA-induced CP (0.3% vs. 2.0%, *p* = 0.027), and acute heart failure/pulmonary edema (0.3% vs. 2.9%, *p* = 0.004) during the procedure were significantly lower in Period II than in Period I. In Period I, a total of 10 CPs occurred in nine patients, with two mechanisms responsible for one CP in one patient. Two CPs occurred because of burr oversizing at the distal and middle LCX in Period I. The majority (60%) of CPs were secondary to bias cutting, including two that occurred in the D1 body, one that occurred in the mid-distal turn of RCA, and another that occurred at the very tortuous mid-LCX. On the other hand, only one Ellis 2 CP secondary to RA occurred in Period II, which could not be attributed to any of the four mechanisms. After reviewing the fluoroscopy and cine recordings, we noted that this CP resulted from the use of the smallest 1.25 mm burr per se. To our surprise, no CPs secondary to wire tip injury, wire transection, burr oversizing, or bias cutting were observed in Period II.

**Table 3:** Procedural outcomes and complications following PCI with RA.

	Period I	Period II	*p*-value
	(2009/1 to 2019/12)	(2020/1 to 2024/2)	
Variables	*N* = 510	*N* = 367	
Acute slow/no flow (*N*, %)	46 (9.0)	18 (4.9)	0.021
Vessel perforation by RA (*N*, %)	10 (2.0)	1 (0.3)	0.027
By burr *per se* (not bias cutting	0 (0)	1 (0.3)	
or oversizing)			
At mid-LCX (no tortuosity)		1	
By wire tip	1 (10)	0 (0)	
At apical LAD	1		
Wire transection	2 (20)	0 (0)	
At mid-LAD (for underexpanded stent)	1		
At LCX-P-M junction (acute angulation)	1		
By burr oversizing	2^a^ (20)	0 (0)	
At distal LCX (not tortuous)	1		
At mid-LCX^a^ (multiple bends)	1		
By unfavorable bias cutting	6^a^ (60)	0 (0)	
At RCA-M-D junction (acute bend)	1		
At D1 body (not tortuous)	2		
At mid-LCX (multiple bends)	1^a^		
At p-LAD (not tortuous)	1		
At RCA-P-M junction (moderate bend)	1		
Vessel perforation by ballooning (*N*, %)	1 (0.2)	2 (0.5)	0.383
Acute heart failure/pulmonary edema (*N*,%)	15 (2.9)	1 (0.3)	0.004
Ventricular arrhythmia (*N*, %)	8 (1.6)	2 (0.5)	0.159
Emergent CABG (*N*, %)	1 (0.2)	0 (0)	0.396
Died on table (*N*, %)	1 (0.2)	0 (0)	0
Acute CIN (*N*/checked, %)^b^	49/173 (28.3)	33/133 (24.8)	0.492
Access hematoma (*N*, %)	11 (2.2)	2 (0.5)	0.052
Rotablation failed	8 (1.6)	6 (1.6)	0.938
Burr stuck	2 (25)	0 (0)	
Could not cross lesion	2 (25)	6 (100)	
Perforation	4 (50)	0 (0)	

CABG, coronary artery bypass grafting; CIN, contrast-induced nephropathy.

a Same patient whose perforation was caused by two mechanisms.

b In selected patients at high risk of acute CIN.

The incidence of rotablation failure was similar between Periods I and II, but this was completely ascribed to failure of the burr to cross the lesion and not to burr stall or any other major complications of RA in Period II.

## 4 Discussion

The major findings of this study were as follows: 1. Modifying RA practice through efforts—including promotion of general awareness of the mechanisms of RA-induced CP, encouraging operator's willingness to stop RA before complications occurred, and provision of case mentorship and support—helped significantly lower the incidence of this dreaded complication at our center, which saw numerous complex PCIs every year. 2. The incidence of acute slow/no flow also declined significantly over time. 3. In spite of complex anatomies with complicated clinical scenarios (including a high percentage of patients requiring hemodynamic support for the procedure) and long procedure times, RA was successfully accomplished in the vast majority of patients, with a very low complication rate. 4. Failure of the burr to cross the lesion remains the major cause of treatment failure in the modern era. This may be attributed to increased cautiousness from the operator for possible RA-related complications and willingness to stop RA before complications occur in difficult lesions.

Treating heavily calcified coronary lesions poses challenges to even the most experienced interventional cardiologists, particularly when lesions involve thick plates or are distributed along acute curvatures. These days, several calcium-modifying tools are available in the catheterization room, including excimer laser, RA, OA, and IVL. Systemic reviews and meta-analyses have demonstrated that IVL achieves angiographic/ procedural success rates comparable to RA, with similar major cardiovascular events but lower risks of perforation and slow/no flow (15). A prospective randomized study showed that IVL had similar procedural success rates as RA but better 1-year safety outcomes after propensity score matching (16). However, in that study, the procedural success rate of both devices was approximately 86%. In another prospective randomized study, IVL and RA showed no differences in minimal stent area, procedural success rates, or complications (8). Again, in that study, the procedural success rate was approximately 93% and crossover to second calcium-modifying device was required in approximately 10.5% of cases. Therefore, IVL seems to supplement but not completely replace RA, especially when it comes to IVL-uncrossable or undilatable lesions. For the most difficult and complex calcified lesions, RA or OA not only paves the way to deliver the IVL catheters but also debulks the thick calcium before the shockwave can take effect, as shockwave catheters may be unable to break very thick calcification plates (17). From an economic perspective, effectively debulking calcium with atherectomy devices could allow balloon dilatation and stent expansion in many lesions, obviating the need for shockwave catheters (18).

RA remains the most popular debulking device for heavily calcified plaques (8) and has been in the market for 38 years since its debut in 1988 (19). Despite its long-standing role in the catheterization laboratory, RA is associated with some major adverse events, particularly in long and complex lesions, and may even result in fatal consequences if not handled properly (20). Current expert consensus emphasizes that slowing the burr advancement rate and exerting minimal advancement force can not only reduce the risk of the burr getting stuck but also that of CP (21). However, the risk of RA-related CP remains a major threat during the procedure, especially in long, complex, and angulated anatomies, and continues to present itself in a low but non-negligible percentage of patients as reported in the literature, including current studies (18, 22–24). CPs are associated with adverse short- and long-term outcomes (25, 26) and represent the dark side of this powerful yet hazardous tool. In 2020, our group published an inspiring paper on the mechanisms of RA-induced CP after a detailed review of fluoroscopy and cine recordings surrounding RA-caused perforation events across a 9-year period. In total, we found four mechanisms of RA-related CP. To our surprise, the most important cause for more than half of the CP cases was unintended and unnoticed RA bias cutting. Since then, we have also noted that almost all the complication cases presented at intervention meetings fall into one of four mechanisms, consolidating the significance and importance of our findings. Therefore, these CP complications may be averted or minimized through structured education, case advice, and case review designed to promote general awareness of these concepts when performing the procedures. We implemented these strategies at our catheterization laboratory and during our meetings, while also mentoring less experienced operators to analyze diagnostic coronary angiograms in terms of three-dimensional distribution of calcification. We also guided less experienced operators through complex RA procedures, emphasizing the use of favorable bias cutting, avoidance of unfavorable bias cutting, and appropriate selection of RotaWire type and burr size. Almost all interventionists adopted these principles and adhered to good practice so as to avoid CP. In our experience, the distribution of thick calcium is best appreciated when the rotablation wire is in place, with a normal frame rate (15 fps), normal radiation setting (not reduced dose setting), and normal FOV being used. With these fluoroscopy settings and the concept of RA bias cutting in mind, a gradual thinning of the calcium plates could be observed by visual inspection after one or few controlled burr runs. RA debulking should be stopped when only minimal thickness of calcium remained and before the burr and wire cutting through the vessel wall as well illustrated in our diagrams in Figure 1. The dramatic reduction in the incidence of RA-induced CP resulted not only from correct application of favorable bias cutting and increased willingness to avoid RA in complex lesions by foreseeing possible complications, but also from avoidance of RA in unfavorable bias cutting cases and minimization of vessel injury by limiting the burr passage through those sites (as illustrated in Figure 2). As expected, our findings in the present study confirmed our hypothesis that structured education, increased general awareness of the mechanisms of RA-induced CP, and mentorship with clinical support can effectively lower the incidence of this feared complication.

Although some differences in baseline demographics (age, distal location, side branches RA) may appear related to the lower incidence of CP in Period II, these factors are most likely reflections of the concept and practice changes implemented in Period II after recognition of the four major mechanisms behind RA-caused CP. As shown in Table 3, two CPs due to burr oversizing occurred at the distal and middle LCX in Period I. The majority (60%) of CPs in Period I were secondary to bias cutting, including two in the D1 body, one in the mid-distal turn of the RCA, and one in the very tortuous mid-LCX. These anatomies predispose to more RA-related to CPs and have to be avoided in very challenging cases. The mean age of the patients experiencing CP was 75.9 years (12), which was significantly higher than the average RA patient age, implying that older patients with complex disease were more prone to opt for PCI but not for CABG. Therefore, less distal and less bifurcation lesions treated by RA and younger patient age did have a relationship to CP, but more likely as a consequence of concept and practice changes in Period II, rather than causing less CP in Period II.

An interesting finding in this study was that, despite the increased operator experience accumulated over time, procedural failures in Period II were exclusively related to failure of burr crossing, whereas perforation-related failures were eliminated. This might also be caused by a less aggressive RA approach as a result of more caution regarding possible RA-related CP following the education. Bias cutting at acute bends, wire transection due to excess stress by repetitive RA attempts before burr crossing, and using RA in relatively small vessels are all important causes for RA-related CP. In our previous study, we recognized that persistence in RA manipulation without awareness of the possible issues accounted for most complications. Therefore, a good attitude based on pragmatic education and mentorship is another important factor contributing to reduced rates of CP.

Another important finding of this study is that we also significantly reduced the incidence of slow/no flow over time. Technically, this was achieved by shortening the duration of each RA run, advancing the burr with gentle force in every single run, promptly managing slow/no flow with high-dose intracoronary adenosine infusion through a dual-lumen microcatheter, and limiting balloon dilatations once slow/no flow occurred during the procedure. Using the smallest 1.25 mm burr and lower serum LDL-C in Period II may have also contributed to the reduced slow/no flow (27). An additional finding of the current study was that RA could be accomplished in a very high percentage of patients as compared to other studies (8, 16), despite complex anatomies and complicated clinical settings (fewer than half of the patients had stable angina, one-third of the patients had CKD, 18.5% had ischemic cardiomyopathy/cardiogenic shock, and Syntax scores were relatively high). The success might be ascribed to increasing experience of operators over time, proper patient preparation, knowledge of how to avoid RA-induced CP and reduce slow/no flow risks, and the use of MCS in a sizable proportion of patients in need. In accordance with standardized CHIP PCI practice, the prophylactic use of MCS in Period II increased significantly, with patients weaned at the end of uneventful procedures. The last finding in our study was that most RA run failures were caused by device (RA burr) uncrossability due to very tight lesions, implying that a new device needs to be invented or the current device needs to be improved to overcome this last challenge.

By obtaining these three major achievements over time, RA has been well adopted and employed at our catheterization laboratories. We suggest that by adopting the same structured education framework to disseminate general awareness of how to avoid RA-induced CP and by offering clinical mentorship, the rate of RA-induced CP could be reduced in other centers as well. Combing with strategies to lower and treat slow/no flow phenomenon and adopting safety principles like using MCS in proper time, good RA practice will promote and ensure patient safety in modern catheterization laboratories.

### 4.1 Limitations

There are several limitations in the present study. First of all, the retrospective study design is subject to inherent biases. Second, this study spanned 15 years, during which changes in periprocedural medications, PCI practices, tools, and operators were inevitable. However, RA devices, peri-RA pharmaceuticals, and practices remained largely unchanged, while senior operators and mentors in our catheterization laboratory oversaw the procedures. What really differed in RA practices between our two study periods was the general awareness of the four mechanisms leading to RA-induced CP and the increased operator caution to stop RA before it caused complications in the second period. Although RA hardware was updated to Rotapro in 2022, the burr, wire, and advancer were basically the same as the old model. Third, although the decision to perform RA was at the discretion of each individual operator, the mentorship and clinical training system at our center were the same over the years, and PCI quality control was well maintained throughout. Only qualified and certified RA operators were allowed to practice RA. Fourth, the incidence of RA-induced CP is low in real-world practice, and improvement by practice may be difficult to detect in small sample sizes. However, the sample sizes in both study periods were reasonably large to allow statistical analysis and to demonstrate clear improvement in outcome after modification of RA practice.

## 5 Conclusions

The implementation of structured education, case advice, and case review to promote general awareness of the four mechanisms of RA-induced CP, together with changes in operator attitude and modification of the real-world practices, helped lower the risk of CP. The key messages conveyed through education and clinical mentoring are as follows: 1. particular caution in tortuous and angulated vessels; 2. gradual burr advancement without excessive forward force and shorter rotational runs in specific scenarios; 3. limiting the number of burr passages once adequate calcium modification has been achieved, with careful evaluation of the adequacy of modification; and 4. operator caution and willingness to stop RA before complications occur. In combination with strategies to lower and treat the slow/no flow phenomenon and adherence to safety principles such as timely use of MCS, good RA practices can more effectively promote and ensure patient safety in modern catheterization laboratories.

## Data Availability

The dataset used for analysis and leading to the study conclusions are readily available. Requests to access the dataset should be directed to Dr. Wen-Lieng Lee, wenlieng.lee@gmail.com.
